# Importance of Prospective Studies in Pregnant and Breastfeeding Women Living With Human Immunodeficiency Virus

**DOI:** 10.1093/cid/ciz121

**Published:** 2019-02-13

**Authors:** Angela Colbers, Mark Mirochnick, Stein Schalkwijk, Martina Penazzato, Claire Townsend, David Burger

**Affiliations:** 1 Department of Pharmacy, Radboud Institute for Health Sciences, Radboud University Medical Center, Nijmegen, The Netherlands; 2 Boston University School of Medicine, Massachusetts; 3 Department of Pharmacology and Toxicology, Radboud Institute for Molecular Life Sciences, Radboud University Medical Center, Nijmegen, The Netherlands; 4 HIV Department, World Health Organization, Geneva, Switzerland; 5 Population, Policy and Practice Programme, UCL Great Ormond Street Institute of Child Health, University College London, United Kingdom

**Keywords:** pregnancy, clinical trials, antiretrovirals, safety, pharmacokinetics

## Abstract

Recently, the US Food and Drug Administration and European Medicines Agency issued warnings on the use of dolutegravir and darunavir/cobicistat for treatment of pregnant women living with human immunodeficiency virus (HIV). It took 3–5 years to identify the risks associated with the use of these antiretroviral drugs, during which time pregnant women were exposed to these drugs in clinical care, outside of controlled clinical trial settings. Across all antiretroviral drugs, the interval between registration of new drugs and first data on pharmacokinetics and safety in pregnancy becoming available is around 6 years. In this viewpoint, we provide considerations for clinical pharmacology research to provide safe and effective treatment of pregnant and breastfeeding women living with HIV and their children. These recommendations will lead to timelier availability of safety and pharmacokinetic information needed to develop safe treatment strategies for pregnant and breastfeeding women living with HIV, and are applicable to other chronic disease areas requiring medication during pregnancy.

## Teaser

The time period between registration of antiretroviral drugs and first pregnancy data becoming available should be shortened. We provide considerations on how to effectively evaluate aspects of clinical pharmacology required for safe and effective treatment to optimize pharmacotherapy during pregnancy and breastfeeding.

In May and June 2018, the US Food and Drug Administration (FDA) and European Medicines Agency (EMA) issued warnings on the use of the antiretroviral agents dolutegravir and darunavir/cobicistat for treatment of pregnant women living with human immunodeficiency virus (HIV). A large observational study detected a 0.9% risk of neural tube defects (NTDs) in infants delivered by women receiving dolutegravir around conception or early in the first trimester of pregnancy. This was considered a substantial risk relative to 0.1% NTDs observed with other antiretrovirals [1–3]. This observation led to a recommendation that dolutegravir should only be used in adolescent girls and women of childbearing potential together with consistent and reliable contraception [4]. The use of the darunavir/cobicistat combination in pregnancy was associated with an average reduction in plasma darunavir concentrations of approximately 50% in pregnancy compared to postpartum, with concentrations in some individual pregnant women reduced by as much as 90% [5]. Low darunavir exposure has been associated with an increased risk of treatment failure and may therefore increase the risk of HIV transmission to the infant [5–7]. Cobicistat and ritonavir levels decrease by approximately 50%–60% during pregnancy, possibly leading to a reduced boosting effect. In the case of boosting with cobicistat, this led to 50% reduction of darunavir exposure (area under the curve [AUC]) and 89% reduction of darunavir minimum concentration (C_min_), whereas the reductions in darunavir exposure and C_min_ when boosted with ritonavir were less remarkable (35% lower AUC and 50% lower C_min_) [7, 8]. In October 2018, this led to FDA label changes for all cobicistat-boosted antiretrovirals, indicating that these should not be used in pregnancy due to substantially lower exposure to the antiretrovirals during the second and third trimesters of pregnancy [9–11].These recent warnings highlight, first, that regulatory authorities take a serious view of the risks for infants of maternal pharmacotherapy during pregnancy. Second, they highlight the importance of collecting safety and pharmacokinetic data on antiretrovirals in pregnant women in a prospective, systematic, and controlled way [3, 12]. Dolutegravir was registered by the FDA in 2013, darunavir/cobicistat in 2015, and elvitegravir/cobicistat in 2012 [13]. Despite the treatment of pregnant women with these agents over the past years, the risks associated with their use during pregnancy were only identified in 2018.

In the past decade, the FDA and EMA have continued to emphasize the need for inclusion of women (pregnant and nonpregnant) in clinical development programs, issuing guidance for industry on how to conduct pharmacokinetic and pharmacodynamic studies in pregnant and lactating women, as well as on establishing pregnancy registries [14–16]. There is still, however, a general lack of legislation or regulations that formally incentivize or mandate drug studies in pregnant women [17].

**Figure 1.**
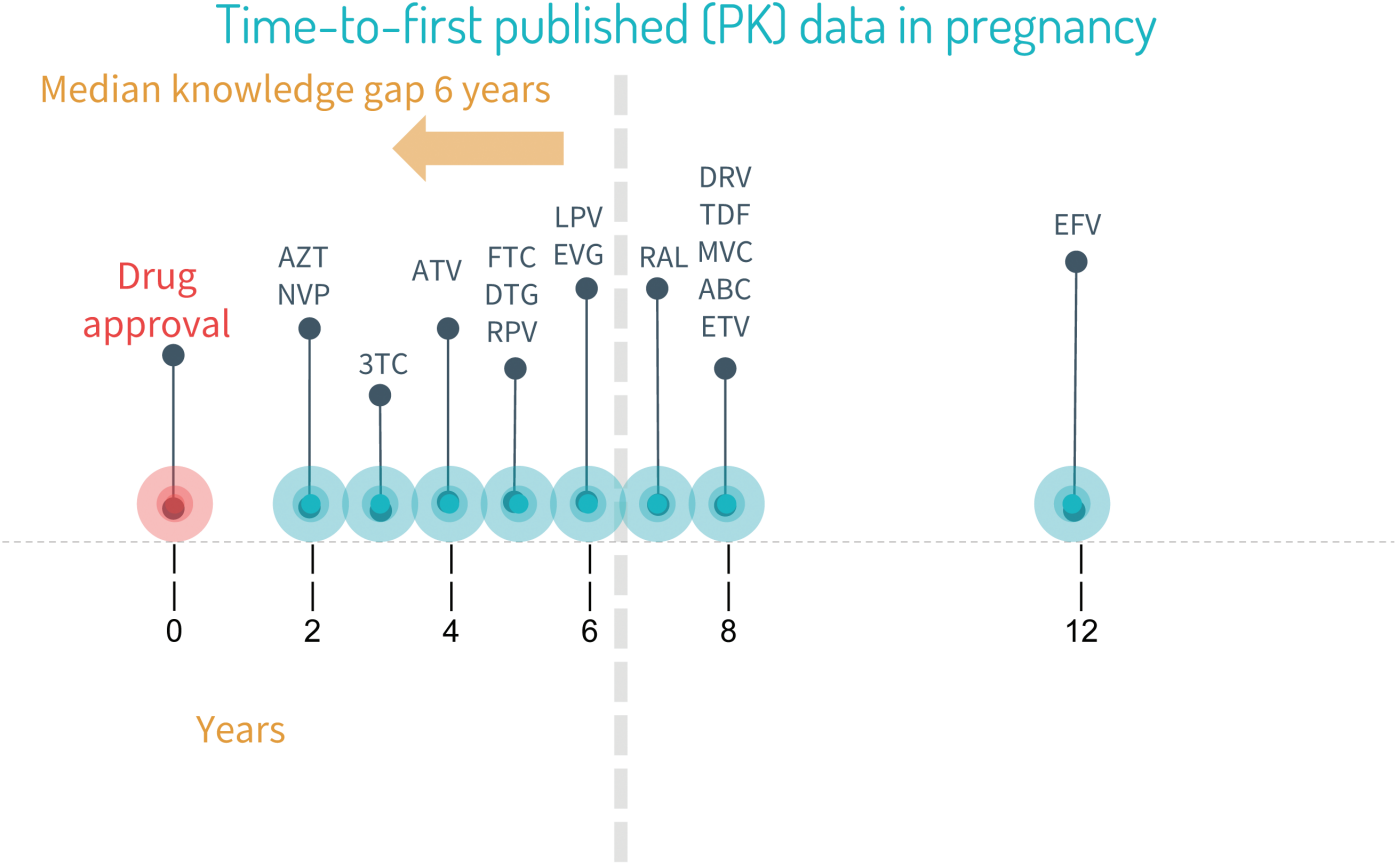
Years between US Food and Drug Administration approval and publication of pregnancy data for different antiretroviral drugs. Abbreviations: 3TC, lamivudine; ABC, abacavir; ATV, atazanavir; AZT, zidovudine; DRV, darunavir; DTG, dolutegravir; EFV, efavirenz; ETV, etravirine; EVG, elvitegravir; FTC, emtricitabine; LPV, lopinavir; MVC, maraviroc; NVP, nevirapine; PK, pharmacokinetic; RAL, raltegravir; RPV, rilpivirine; TDF, tenofovir disoproxil fumarate.

Despite efforts to promote postmarketing surveillance and investigate the pharmacokinetics and safety of antiretroviral agents during pregnancy [18–20], data from these studies usually become available years after approval by stringent regulatory authorities (Figure 1). As a result, a substantial number of pregnant and breastfeeding women will inevitably use antiretroviral agents in the absence of any pregnancy-specific safety or pharmacokinetic data, putting both mother and infant at potential risk. Healthcare professionals are faced with the difficult balancing act of either accepting the potential risks of using newer antiretrovirals despite the absence of safety and pharmacokinetic data or denying pregnant and lactating women access to antiretrovirals that may offer significant benefits over older agents [21]. Consequently, there is a need for information, gathered under rigorous scientific conditions, on antiretroviral pharmacokinetics, efficacy, and safety during pregnancy and lactation [22]. Nevertheless, there are a number of important considerations when performing clinical studies in pregnant women. It is important that these are acknowledged and that existing knowledge gaps are addressed where possible.

In July 2018, the World Health Organization (WHO) Paediatric Antiretroviral Working Group launched a toolkit for research and development of paediatric antiretroviral drugs and formulations, which aims to guide industry on approaches for accelerating the development of antiretrovirals for children and pregnant and lactating women [23]. In this toolkit, the working group provides considerations for effectively evaluating all aspects of clinical pharmacology that are required for safe and effective treatment of women living with HIV and their children and to optimize pharmacotherapy during pregnancy (Table 1). This article touches on the issues raised in the specific pregnancy module of the toolkit and expands on these issues.

**Table 1.** Considerations for Clinical Pharmacology Research in Pregnancy

1. Placental transfer should be studied during the preclinical phases of drug development using techniques such as in vitro–in vivo extrapolations or ex vivo human cotyledon perfusion models.
2. Regulatory authorities and ethics committees should incentivize and support inclusion of pregnant women in premarketing clinical trials for compounds potentially being used in pregnancy. As a first step, women enrolled in phase 2 or phase 3 clinical trials should not be removed from the study drug if they become pregnant during the trial.
3. Antiretroviral clinical pharmacology studies in pregnant and lactating women should be executed according to the highest standards and requirements.
4. Modeling and simulation should be used to facilitate understanding of pregnancy-related clinical pharmacology and inform clinical studies in pregnant women.
5. Cord blood samples and maternal samples should be taken at delivery to assess fetal exposure, and blood samples in the neonate should be taken to assess neonatal elimination.
6. Postpartum lactating women should be included in clinical trials and breast milk transfer from mother to infant should be assessed.
7. Safety of antiretroviral therapy and pregnancy outcomes should be closely monitored during pharmacokinetic studies that include pregnant women and in (obligatory reporting in) postmarketing surveillance studies, preferably in a global database.

## Ethical Concerns Regarding Exposure of Pregnant Women and Their Fetuses to Antiretroviral Drugs Under Development

Although there are clearly ethical considerations when including pregnant and breastfeeding women in clinical trials, it can also be considered unethical not to test new drugs in pregnant women in a controlled setting. In practice, once new drugs are approved in adults, their use rapidly expands to include pregnant and breastfeeding women, exposing more mothers and infants to these potential risks than would have occurred in the context of a clinical trial.

## Birth Defects and Other Adverse Birth Outcomes

Essential elements of pregnancy-related clinical pharmacology are the direct and indirect drug effects on the fetus. Indirect drug effects, such as increased risk of preterm labor or impaired glucose homeostasis, may have profound effects on fetal well-being. Also, many drugs cross the placenta, resulting in exposure of the fetus, with potential toxicity. Exposure during the first trimester may impact fetal organogenesis and result in teratogenicity. Exposure later in pregnancy may put the fetus at risk for impairments of growth and development or harm to specific organ systems. Fetal exposure to antiretroviral drugs may also provide beneficial effects such as preexposure prophylaxis that may aid in preventing perinatal HIV transmission [24]. Short- and longer-term monitoring of infants exposed to antiretrovirals in utero is essential, but many pregnancy registries are based on voluntary reporting, which results in bias and often leads to reporting delays.

## Physiological Changes in Pregnancy May Affect Exposure to Antiretroviral Drugs

Pregnancy is associated with a wide range of physiological, anatomical, and biochemical changes that substantially impact the pharmacokinetics of therapeutic agents [25, 26]. Prolonged gastric transit time, nausea and vomiting, and dietary alterations may alter drug absorption. Drug distribution in pregnant women may change because of changes in body composition, blood volume, protein binding, and expression of transporters. Activity of drug-metabolizing enzymes may increase (eg, CYP3A, UGT1A4) or decrease (eg, CYP2C19), affecting the intrinsic clearance of antiretroviral agents. Increases in cardiac output, renal blood flow, and glomerular filtration rate may increase elimination of renally cleared drugs. In combination, these changes may result in alterations of the unbound pharmacologically active concentration of drug at the target site, leading to changes in drug response. For some antiretrovirals, such as lopinavir/ritonavir, darunavir/ritonavir, elvitegravir/cobicistat, and darunavir/cobicistat, these changes have such an impact on drug exposure during pregnancy that pregnancy-specific dosing recommendations have been developed; in some cases, the drugs are not recommended for use in pregnancy. Furthermore, drugs and vitamins typically used during pregnancy may interact with antiretrovirals on an enzyme level, but also on transporter levels (ie, iron tablets and dolutegravir). Studying the pharmacokinetics of antiretroviral drugs in pregnant women and understanding their interactions with other commonly used drugs or supplements is necessary to ensure adequate drug exposure in this vulnerable population and also to be able to assess the possible transfer of drugs over the placenta and into breast milk.

## Placental Transfer, Fetal Exposure, and Disposition Into Breast Milk Are Unknown

Physiologically, placental transfer is the main determinant of fetal exposure during pregnancy [27]. However, quantifying fetal exposure in humans is not straightforward, as the fetus itself is not accessible for sampling during pregnancy. Assessment of fetal drug exposure is usually limited to cord blood sampling at the time of delivery.

After delivery, infants can also be exposed to antiretrovirals through breast milk. Transmission of HIV from mother to child in the postnatal period via breast milk remains a major contributor to perinatal transmission rates in low- and middle-income countries, where women commonly breastfeed for ࣙ2 years. The provision of maternal antiretroviral therapy during the breastfeeding period has been shown to significantly reduce breast milk HIV transmission, by reducing breast milk HIV concentrations and/or by providing prophylaxis to the infant through ingestion of antiretroviral drugs present in breast milk [28]. However, antiretroviral exposure during breastfeeding could result in drug-related toxicity in the infant or the development of drug-resistant HIV strains in HIV-infected infants who receive subtherapeutic drug exposure via breast milk. Clinical pharmacology studies in this field are still very limited [29].

## Recommendations

Regulatory authorities and ethics committees should incentivize and support inclusion of pregnant women in premarketing clinical trials for compounds with potential for use in pregnancy. Ethically acceptable strategies for conducting research on HIV treatment and prevention during pregnancy are being studied in the Pregnancy and HIV/AIDS: Seeking Equitable Study (PHASES) project [30]. PHASES will focus on 3 specific areas of HIV research in pregnant women: prevention, treatment of coinfection, and disease management. Based on an analysis of HIV-related trials involving pregnant women reported in the International Clinical Trials Registry Platform database, the PHASES group concluded that research in pregnant women in the context of HIV is conducted with some frequency and through a variety of sponsors, locations, and study designs. However, important opportunities for improving the evidence base remain, including advancing research on HIV prevention strategies for pregnant women and investigating safety and efficacy of study compounds during pregnancy earlier in the drug development cycle [31].

Dedicated clinical pharmacology studies in pregnant and lactating women can be initiated once initial phase 2 safety and efficacy data have been demonstrated in nonpregnant adults. These studies could include the women from premarketing trials and continue to include more pregnant women for adequate power with respect to a prespecified clinical endpoint, for example, undetectable viral load at delivery. The studies may be opportunistic (ie, patients who become pregnant while receiving a specific drug can be included without changing treatment) or interventional (ie, studies in pregnant women conducted to search for the optimal dose), and may be performed by academia, the pharmaceutical industry, or a collaboration between both. To accelerate inclusion rates and include women in the setting where the disease is most prevalent, these studies should be performed in relevant populations in both high-income and low- and middle-income country settings. Centers of excellence should be established in low- and middle-income countries.

Antiretroviral clinical pharmacology studies in pregnant and lactating women should be executed according to the highest standards and requirements. These studies should include intensive pharmacokinetic sampling over a dosing period in the third trimester of pregnancy (minimum), second and first trimesters (if possible), and a postpartum reference curve for intrasubject comparison (at least 4 weeks postpartum). Furthermore, random sampling during the entire pregnancy to support population pharmacokinetic modeling is highly recommended. Unbound plasma concentrations should be determined for highly protein-bound drugs (>80% bound). Collection of cord and maternal blood samples at delivery will allow assessment of placental transfer of the drugs in vivo. Antiretroviral infant washout samples following delivery should be collected, especially for drugs with a long half-life and those metabolized by enzymes not fully developed in newborns (eg, uridine 5′-diphospho-glucuronosyltransferase). These pharmacology studies should also collect efficacy and safety data, and should be included in global pregnancy registry databases.

Postpartum lactating women should be included in clinical trials to allow assessment of breast milk transfer from mother to infant. Breast milk sampling can be done randomly during the 3 phases: colostrum (starts secreting around 24 weeks of gestation), transitional (days 2–3 postpartum), and mature milk phase (days 10–14 postpartum). To assess transfer rates into breast milk, maternal blood, breast milk, and infant blood should be collected at around the same time randomly after medication intake. Collection of full curves (as proposed in the FDA [draft] guidance for clinical lactation studies) may not be necessary [32].

Placental transfer should be studied during the preclinical phases of drug development using techniques such as in vitro–in vivo extrapolations or ex vivo human cotyledon perfusion models. This will lead to early indications of whether drugs are likely to transfer through the placenta and reach the infant at potentially toxic levels. Such studies will require cooperation between academia and the pharmaceutical industry, as perfusion models are typically set up in academic hospitals, due to the limited time window between collection of placentas and start of perfusion.

Furthermore, basic and clinical research exploring the mechanisms (ie, enzyme/transporter expression) of the pregnancy effect on maternal drug exposure, as well as the mechanisms behind transfer of drugs across the placenta and into breast milk, is needed to make physiologically based pharmacokinetic models of pregnancy more accurate and robust.

Although safety of antiretroviral therapy and pregnancy outcomes should be closely monitored during pharmacokinetic studies that include pregnant women, pharmacovigilance with postmarketing pregnancy surveillance studies, preferably global in scope, will still be necessary to delineate low-frequency adverse pregnancy outcomes. If all pregnancy outcomes of the first exposures to drugs during pregnancy are registered (>1000 as an indication), safety signals may be picked up at an early stage after licensure, without delaying access to the new compounds for women of childbearing potential.

Inadequate drug exposure with the use of cobicistat-boosted drugs in pregnancy could have been recognized earlier if pregnancy pharmacokinetic studies had been performed at an earlier stage of drug development or if outcomes of initial pregnancies exposed after licensure had been registered. Regulatory requirements for obligatory studies in pregnancy should not delay the registration process or the availability of new drugs for women of childbearing potential, if these studies are routinely incorporated into the drug development process. Regulatory incentives may help successfully ease the transition to performance of initial pregnancy pharmacology studies as part of drug development and obligatory registration of pregnancy outcomes after licensure.

An approach based on the considerations outlined here and in the WHO toolkit for research and development of pediatric antiretroviral drugs and formulations will facilitate the timelier availability of safety and pharmacokinetic information needed to develop safe treatment strategies for pregnant and breastfeeding women living with HIV. This approach is also applicable to other chronic disease areas where medication during pregnancy is required, such as diabetes mellitus, epilepsy, and autoimmune diseases. Cross-sector efforts are needed to accelerate research and ensure that protecting women and their babies does not delay their access to universal health coverage.

## Notes

***Acknowledgments.***The authors thank Gerhard Theron, Alice Stek, and Lynne Mofenson for review of the module on pregnant and breastfeeding women of the toolkit for research and development of pediatric antiretroviral drugs and formulations, and the members of the World Health Organization Paediatric Antiretroviral Working Group (PAWG) for their input.

***Potential conflicts of interest.*** A. C. and M. M. received a grant for their contribution to the PAWG toolkit for research and development of pediatric antiretroviral drugs and formulations. M. M. has received personal fees from the International Maternal Pediatric Adolescent AIDS Clinical Trials Network, grants from Gilead, and grants and personal fees from ViiV. A. C. has received grants to her institution from PENTA, ViiV, Janssen Research, Gilead, and Merck. All other authors report no potential conflicts. All authors have submitted the ICMJE Form for Disclosure of Potential Conflicts of Interest. Conflicts that the editors consider relevant to the content of the manuscript have been disclosed.
